# Causal effects of genetically vitamins and sepsis risk: a two-sample Mendelian randomization study

**DOI:** 10.1186/s12879-023-08778-9

**Published:** 2023-11-07

**Authors:** Chen Lou, Zhizhen Meng, Yiyi Shi, Rui Zheng, Jingye Pan, Songzan Qian

**Affiliations:** 1 School of The First Clinical Medical Sciences, Wenzhou Medical University, Wenzhou, 325000 China; 2 Department of Emergency, The Fourth Affiliated Hospital, Zhejiang University School of Medicine, Yiwu, China; 3 Department of Anesthesiology, The First Affiliated Hospital of Wenzhou Medical University, Wenzhou, 325000 China; 4 Department of Critical Care Medicine, Sir Run Run Shaw Hospital, School of Medicine, Zhejiang University, Hangzhou, Zhejiang 310016 China; 5 Department of Intensive Care Unit, The First Affiliated Hospital of Wenzhou Medical University, Wenzhou, 325000 China; 6 Wenzhou Key Laboratory of Critical Care and Artificial Intelligence, Wenzhou, China; 7 Key Laboratory of Intelligent Treatment and Life Support for Critical Diseases of Zhejiang Provincial, Wenzhou, Zhejiang 325000 People’s Republic of China; 8 Zhejiang Engineering Research Center for Hospital Emergency and Process Digitization, Wenzhou, Zhejiang 325000 China

**Keywords:** Vitamins, Sepsis, Critical care, Mendelian randomization, Genome-wide association study

## Abstract

**Background:**

In recent years, observational studies have been conducted to investigate the potential impact of vitamins on sepsis. However, many of these studies have produced inconsistent results. Our Mendelian randomization (MR) study aims to evaluate the causality between vitamins and sepsis from a genetic perspective.

**Methods:**

Our MR study was designed following the STROBE-MR guidelines. Genetic instrumental variables for vitamins including folate, vitamin B12, B6, A (Retinol), C, D, and K were obtained from previous genome-wide association studies (GWAS) and MR studies. Five different sepsis severity levels were included in the analysis. The genetic instrumental variables were screened for potential confounders using PhenoScanner V2. MR analysis was performed using MR-egger, inverse-variance weighted multiplicative random effects (IVW-RE), inverse-variance weighted multiplicative fixed-effects (IVW-FE), and wald ratio methods to assess the relationship between vitamins and sepsis. Sensitivity analysis was performed using the MR-egger_intercept method, and the MR-PRESSO package and Cochran’s Q test were used to evaluate the heterogeneity of the instrumental variables.

**Results:**

Our MR study found no statistically significant association between vitamins and sepsis risk, regardless of the type of vitamin (P-value > 0.05). The odds ratios (ORs) for folate, vitamin B6, vitamin B12, vitamin A, vitamin D, vitamin K, and vitamin C were 1.164 (95% CI: 0.895–1.514), 0.987 (95% CI: 0.969–1.005), 0.975 (95% CI: 0.914–1.041), 0.993 (95% CI: 0.797–1.238), 0.861 (95% CI: 0.522–1.42), 0.955 (95% CI: 0.86–1.059), and 1.049 (95% CI: 0.911–1.208), respectively. Similar results were observed in subgroups of different sepsis severity levels.

**Conclusions:**

Our MR study found no evidence of a causal association between vitamins and sepsis risk from a genetic perspective. Further randomized controlled trials are necessary to confirm these results.

**Supplementary Information:**

The online version contains supplementary material available at 10.1186/s12879-023-08778-9.

## Introduction

As the most common trigger of infection-related death, sepsis is the result of an unbalanced host immune response to infection [1]. Sepsis is often accompanied by persistent inflammation, organ failure, and vascular endothelial and mitochondrial dysfunction [2]. There were 11 million sepsis-related deaths in the world in 2017, which made up 19.7% of all fatalities [3]. Early broad-spectrum antimicrobial therapy is considered crucial [4] and general critical care also play an important role [5] in treating sepsis.

Vitamins are essential micronutrients in the human body. It has been widely reported that vitamins can prevent infections, reduce inflammation, and increase antioxidant activity [6]. Thus, adjuvant vitamin therapy in critical illness like sepsis is getting more and more attention, especially for vitamin C and vitamin D [7]. Related randomized controlled trials have been conducted, but results have been inconsistent. It is well known that vitamin C (ascorbic acid) has powerful antioxidant properties. However, researchers found vitamin C to be risky for patients with sepsis in a recent study. Within 28 days, patients who with sepsis receiving vasopressor therapy in the ICU are more likely to die or experience organ dysfunction after intravenous vitamin C injection [8]. This result is different from previous studies and needs more verification. Vitamin D also helps the body’s immune system and suppress inflammation. But different studies had yielded heterogeneous results [9]. Apart from vitamin C and D, other vitamins may also have potential association with sepsis and changes in the concentrations of many vitamins can be observed in people with sepsis. According to a study, children with sepsis are more likely to be vitamin A deficient, which may lead to a higher mortality rate [10]. But the effectiveness of vitamins in treating sepsis remains controversial.

Therefore, unlike previous randomized controlled trials, in our study, the causality between vitamins and sepsis is explained genetically. Our study included five different levels of sepsis and a variety of common vitamins, including B vitamins (folate, vitamin B12, vitamin B6), Vitamin A(Retinol), Vitamin C, Vitamin D, Vitamin K. Confounders and reverse causality can be problematic in traditional observational studies. And these studies have high requirements for implementation conditions. In our study, we use mendelian randomization to study the causal effects between vitamins and sepsis. Mendelian randomization is based on Mendelian laws of heredity. Parents’ alleles are randomly assigned to their offspring in natural conditions, a process similar to randomization in randomized controlled trials, but without environmental confounders factors.

## Methods

### Mendelian randomization study design

**Fig. 1:**
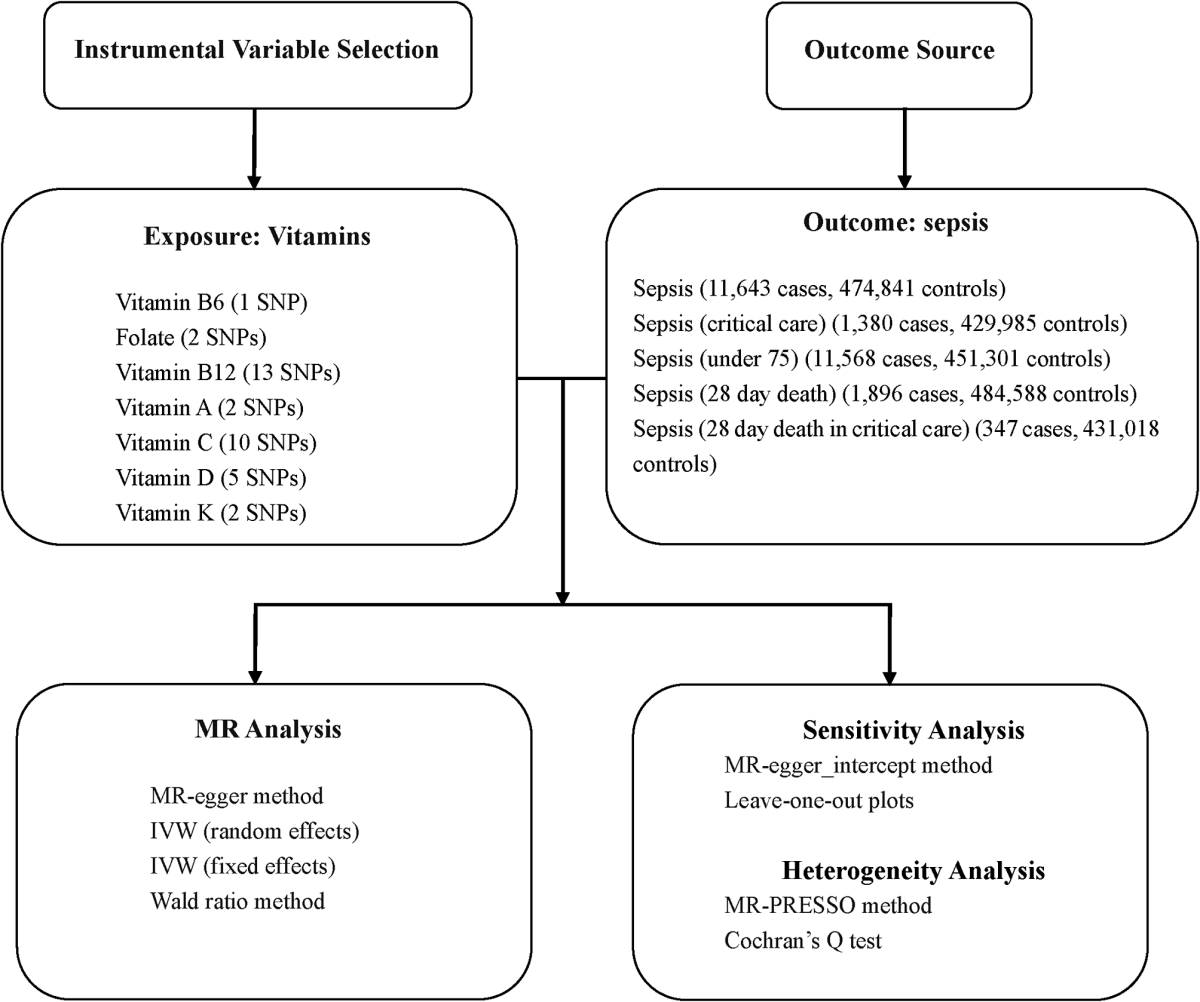
The flow chart shows the content and process of our research

This study is designed according to the STROBE-MR guidelines [11] to make sure that we’ve done every step of Mendelian randomization analysis. Our complete study design is shown in Fig. 1. An MR study with two samples is conducted to examine the causality between genetically vitamins and sepsis risk. It is the first study to use Mendelian randomization to analyze the effect of vitamins on sepsis of different severity from a genetic perspective. What’s more we also analyzed the effect of sepsis on plasma vitamin C levels.

Mendelian randomization studies need to follow three assumptions. In analyzing the effects of vitamins on different levels of severity, assumption 1 is that instrumental variables (IVs) are strongly associated with exposure, namely different vitamins. Assumption 2 is that IVs can only be related to exposure factors, not confounders factors (such as age, sex, BMI, etc.). Assumption 3 is that SNP can only affect the outcome(sepsis) through vitamins, not through other pathways. The study of the effects of sepsis on plasma vitamin C levels also follows the three assumptions described above.

### Data source

In this study, we have selected genetic instrumental variables from previously conducted genome-wide association studies (GWAS) and Mendelian Randomization (MR) analyses, focusing mainly on vitamins that have been researched in the context of infectious diseases. We ensured that the vitamins included are backed by publicly accessible GWAS data, thus facilitating the MR analysis. It is important to note that, due to the inability to represent external vitamin intake or injections through genetic instrumental variables, our approach hinges on using GWAS data derived from the concentrations of vitamins measured in circulating blood. SNPs of B vitamins (folate, vitamin B12, vitamin B6) are selected from a previous mendelian randomization study [12]. The SNPs estimated 1. 0% of heritability for folate, 1. 3% of heritability for vitamin B6 and 6. 0% of heritability for vitamin B12 [12]. SNPs of vitamin A (circulating retinol levels) are selected from a GWAS study, which includes two cohorts of men with 5006 Caucasian individuals [13]. SNPs of vitamin C (plasma vitamin C levels) are selected from a meta-analysis of EPIC-Norfolk, Fenland, EPIC-InterAct, and EPIC-CVD study with a total of 52, 018 individuals [14]. Instrumental variables of vitamin D (25-hydroxyvitamin D serum concentrations) are extracted from a large-scale GWAS meta-analysis with 79, 366 individuals, which explained 7. 5% variance for vitamin D [15]. SNPs of vitamin K (circulating phylloquinone concentrations) are extracted from a Meta-analysis of GWAS studies [16]. Only 2 unrelated SNPs of vitamin K are selected after removing the highly correlated SNPs, SNPs strongly related to lipids and SNPs not in our outcome data.

The GWAS data for sepsis of different severity were derived from a reanalysis of the UK Biobank data in 2021 [17]. Our study included sepsis of varying severity, including Sepsis, Sepsis (critical care), Sepsis (under 75), Sepsis (28-day death), Sepsis (28-day death in critical care). For those traits, the unit is logOR. The sex includes males and females. The population is European. These septic traits were used as outcome of vitamin exposures and as exposure of plasma vitamin C levels outcome.

All study populations and cohorts were European populations. This ensures that the study is carried out in a homogeneous population, satisfying the Hardy-Weinberg law of population inheritance. Detailed information as well as related articles are listed in the Table 1.

**Table 1:** Data source and detailed information of vitamins and different severity of sepsis

Trait	Number of SNPs used	Source	Sample size	Population
Vitamin B6	1	10. 1186/s12916-021-01977-8	37, 465	European
Folate	2	10. 1186/s12916-021-01977-8	45, 576	European
Vitamin B12	13	10. 1186/s12916-021-01977-8	1, 864	European
Vitamin A	2	10. 1093/hmg/ddr387	5, 006	European
Vitamin C	10	10. 2337/dc20-1328	52, 018	European
Vitamin D	5	10. 1038/s41467-017-02662-2	79, 366	European
Vitamin K	2	10. 3945/ajcn. 114. 093146	2, 138	European
Sepsis	8	UK Biobank	486, 484	European
Sepsis (critical care)	4	UK Biobank	431, 365	European
Sepsis (under 75)	3	UK Biobank	462, 869	European
Sepsis (28-day death)	14	UK Biobank	486, 484	European
Sepsis (28-day death in critical care)	5	UK Biobank	431, 365	European

### Genetic instrumental variable selection

We use TwoSampleMR R package (version 0. 5. 6) to choose suitable SNPs. Instrumental variables require a strong correlation with exposure (p < 5 × 10^− 8^). When the exposures are vitamins, the above filter condition can be met, while when sepsis is exposure, the above filter condition (p < 5 × 10^− 8^) cannot be met. Therefore, the weaker filter condition (p < 5 × 10^− 6^) is used to select the instrumental variables of sepsis. Moreover, these SNPs with the linkage disequilibrium (LD)need to be removed to ensure that they inherit independently (r2 = 0.001, kb = 10,000) from each other. Palindromic SNPs that may cause strand ambiguity are also removed. In addition, in order to satisfy assumption 2, 3, we searched the SNPs already obtained through PhenoScanner V2 (http://www.phenoscanner.medschl.cam.ac.uk/) [18] to find potential confounders and bypassing (e.g. Age, sex, race or ethnicity, underlying disease) [2] that might influence the association between vitamins and sepsis. SNPs associated with confounders are removed. Then we calculate F statistics (F = beta^2^/se^2^) [19] for each SNP to evaluate SNP’s statistical power. SNPs with inadequate statistical power (F < 10) were excluded.

### Mendelian randomization analysis

The TwoSampleMR R package (version 0. 5. 6) was used to perform the mendelian Randomization(MR) analysis. For exposures with three or more SNPs, we chose three methods including MR-egger, inverse-variance weighted multiplicative random effects (IVW-RE) and inverse-variance weighted multiplicative fixed-effects (IVW-FE) to evaluate the association between vitamins and sepsis. For exposures with two SNPs, only the latter two methods are applicable. For exposures with one SNP, we adopted the Wald ratio method to conduct MR. MR analysis yielded a P-value to assess whether there was an association between vitamins and sepsis and an odds ratio(OR) to assess the extent to which exposure affected the outcome. The P-value should be less than 0. 05 to indicate a significant association. For the selection of the above MR Methods, we need to conduct sensitivity and heterogeneity analysis for the acquired SNPs. Sensitivity analysis was conducted using MR-egger_intercept method when the number of SNPs of exposure was greater than three [20], and P-value < 0. 05 is considered pleiotropic. The leave-one-out plots is also used to observe differences in the effect of SNPs. SNPs with great different effects were excluded. For heterogeneity analysis, the MR-PRESSO package [20] was used to identify SNPs with heterogeneity and those SNPs was removed. Cochran’s Q test was also used to assess the heterogeneity of SNPs [21]. Cochran’s Q test includes MR-egger method and inverse variance weighted method. Significant heterogeneity was defined as a P-value less than 0. 05. If the heterogeneity is significant, IVW-RE method is adopted to adjust the heterogeneity; otherwise, IVW-FE method is adopted.

## Results

When the exposures are vitamins and outcome was sepsis, we extract one SNP for vitamin B6, two SNPs for folate, thirteen SNPs for vitamin B12, two SNPs for vitamin A, ten SNPs for vitamin C, five SNPs for vitamin D, two SNPs for vitamin K. We have removed the SNPs that does not satisfy the selection criteria that we gave earlier. Table S1 showed detailed information (beta, SE, F statistics) of the SNPs selected for vitamins.

After previous adjustment of possible confounders, in the sensitivity test, no group in MR Test showed pleiotropy (Table S4). In the heterogeneity analysis, MR-presso analysis of each group did not find SNPs with significant heterogeneity. However, in our Cochran’s Q test (Table S4), there were three groups with great heterogeneity. Vitamin D is the exposure and Sepsis is the outcome (Q(Q_pval) = 12.379(0.015)). Vitamin D is the exposure and Sepsis (under 75) is the outcome (Q(Q_pval) = 10.573(0.032)). Vitamin C is the exposure and Sepsis (28 day death) is the outcome (Q(Q_pval) = 17.186(0.046)). For these three groups we apply IVW-RE method to Mendelian Randomization Analysis. The vitamin B6 exposure group with only one SNP had taken the Wald ratio method to conduct MR. All the other groups were analyzed by IVW-FE method.

In Mendelian Randomization Analysis studying the association between vitamins and sepsis risk, the effect of vitamins on sepsis was not statistically significant regardless of different kinds of vitamins and the severity of sepsis outcome (P-value > 0. 05, OR (95% confidence intervals) crossed the null line).

**Fig. 2:**
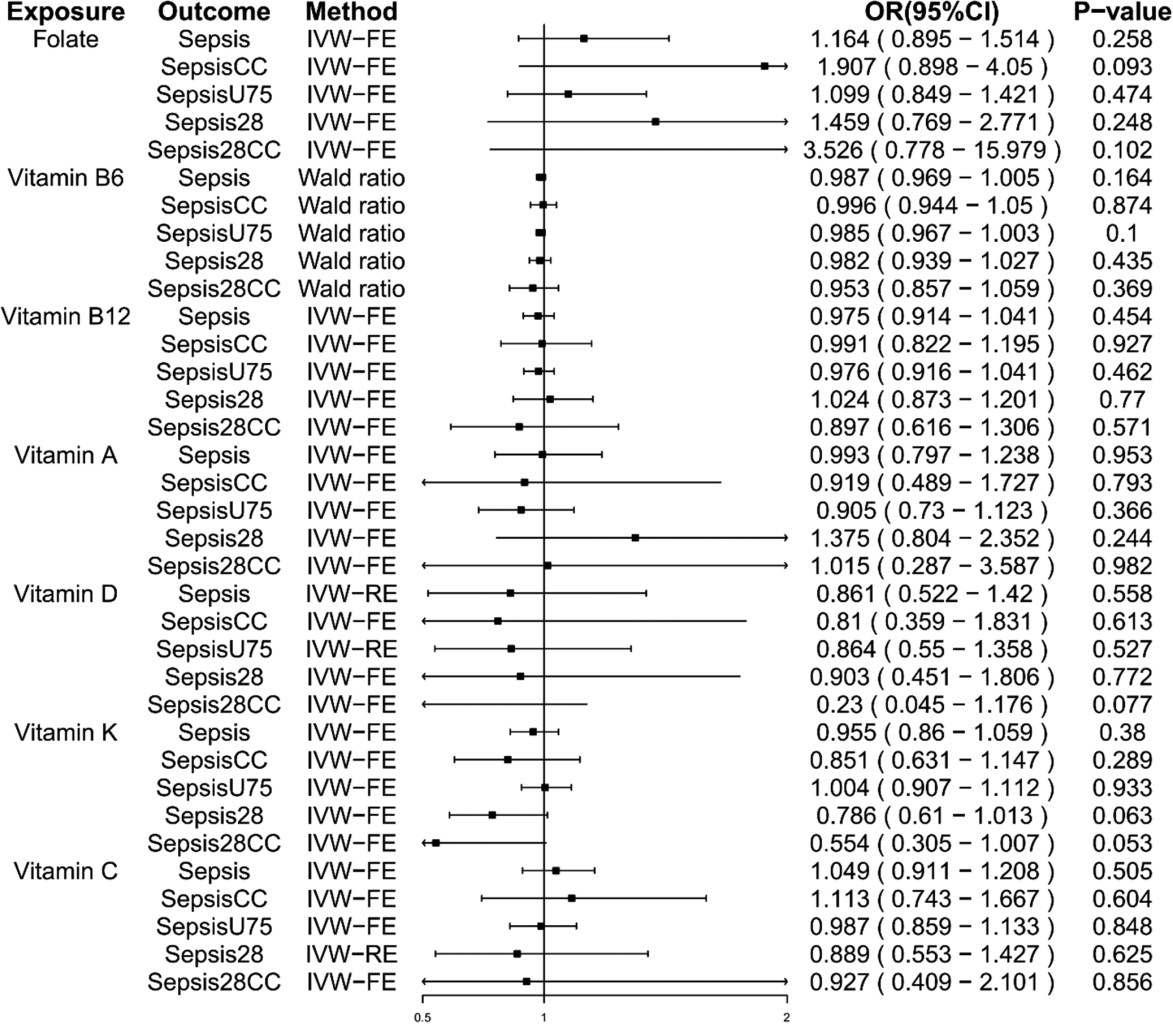
Forest plot illustrates causal effects of vitamins on sepsis outcomes. IVW-RE, inverse-variance weighted multiplicative random effects; IVW-FE, inverse-variance weighted multiplicative fixed-effects; OR (95%CI), odds ratio (95% confidence interval); SepsisCC, Sepsis (critical care); SepsisU75, Sepsis (under 75); Sepsis28, Sepsis (28- day death); Sepsis28CC, Sepsis (28 day death in critical care)

The results showed that the concentration changes of vitamin B6, B12, A, D, C, K and folate had no significant effect on the improvement of sepsis of different severity, The ORs (odds ratio) are 1.164 (95% CI:0.895–1.514; folate),0.987 (95% CI: 0.969–1.005; vitamin B6), 0.975 (95% CI: 0.914–1.041; vitamin B12), and 0.993 (95% CI: 0.797–1.238; vitamin A) 0.861(95% CI:0.522–1.42;D); 0.955( 95% CI:0.86–1.059; vitamin K); 1.049(95% CI: 0.911–1.208; vitamin C) respectively; Similar results were seen in subgroups for different severity sepsis. Details in Fig. 2 and Table S4.

**Fig. 3:**
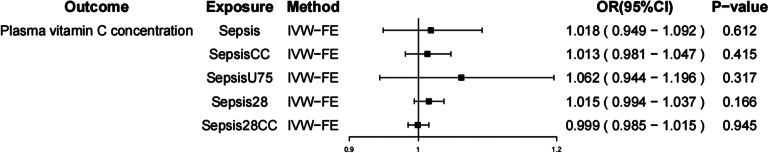
Forest plot illustrates causal effects of sepsis on plasma vitamin C concentration outcomes. IVW-RE, inverse-variance weighted multiplicative random effects; IVW-FE, inverse-variance weighted multiplicative fixed-effects; OR (95%CI), odds ratio (95% confidence interval); SepsisCC, Sepsis (critical care); SepsisU75, Sepsis (under 75); Sepsis28, Sepsis (28 day death); Sepsis28CC, Sepsis (28 day death in critical care)

In another set of mendelian randomization analysis, we investigated whether sepsis of different severity will cause changes in vitamin C concentration. The exposures are different severity of sepsis. We selected ten SNPs for Sepsis, four SNPs for Sepsis (critical care), five SNPs for Sepsis (under 75), fourteen SNPs for Sepsis (28 day death), seven SNPs for Sepsis (28 day death in critical care) at beginning (Table S2). And then several SNPs that were strongly associated with confounders were excluded. For example, rs114920411 is significantly associated with rheumatoid arthritis (P-value = 3. 70E-30) and was excluded. All the SNPs related to confounders were listed in Table S3. And the outcome is plasma vitamin C levels [14]. Our result showed that the ORs (odds ratio) are 1.018 (95% CI: 0.949–1.092; Sepsis), 1.013 (95% CI: 0.981–1.047; SepsisCC), 1.062 (95% CI: 0.944–1.196; SepsisU75), and 1.015 (95% CI: 0.994–1.037; Sepsis28) 0.999 (95% CI: 0.985–1.015;Sepsis28CC) respectively. Sepsis of different severity had no significant association with changes of vitamin C concentration (Fig. 3, Table S5).

## Discussion

In this Mendelian Randomization Study, we explored causal relationship of genetically vitamins and sepsis risk and whether sepsis has an effect on changes in vitamin C concentration in the body. Our findings suggest that vitamins were not associated with sepsis risk or improvement or deterioration of sepsis of any severity. Sepsis had no influence of vitamin C concentration. Compared to previous studies, our Mendelian randomization study is the first to use instrumental variables to represent different severity of sepsis and the first to look at the causality between vitamins and sepsis risk genetically. There have been many studies on the effects of vitamins on sepsis, but whether vitamins have a beneficial effect on sepsis is still controversial and remains to be discussed.

Over the last few years, many randomized controlled trials have been conducted to examine whether vitamin C and vitamin D have direct therapeutic effects on sepsis. Many studies have produced contradictory results, however. Sepsis patients who received Intravenous vitamin C injection for more than five days were significantly less likely to require hospitalization and to die within 90 days [22]. Our study and others in the past suggest that vitamins have no effect on sepsis [23]. During 30 days in patients with sepsis, vitamin C did not increase ventilator-free days or vasopressor-free days by a significant amount [24]. Infusion of vitamin C for 96 h also fail to enhance scores for organ dysfunction and reduce damage to the vascular system and inflammatory markers [25]. We provide genetic evidence that vitamin C does not affect sepsis. As for vitamin D, there is some evidence that suggests insufficient vitamin D can lead to a worsening of sepsis. There was an association between inadequate vitamin D and higher risk of severe sepsis in patients admitted with a possible infection to the emergency department [26]. A high-dose vitamin D supplement reduced 28-day mortality in patients with vitamin D deficiency who were critically ill [27]. However, in another study, the similar supplement given to patients could not decrease hospital stay, in-hospital mortality, six-month mortality [28] and showed no advantage over controls in 90-day mortality and other related outcomes [29] in vitamin D-deficient patients in critical condition. In a previous mendelian randomized study, vitamin D was also found to have no therapeutic value for sepsis [30]. Our MR Study replaces the sources of vitamin D exposure and sepsis outcome, but also supports this idea. Previous randomized controlled trials have produced many different results, possibly due to confounders and different time of vitamin administration. To solve this problem, we included cases of sepsis, cases of sepsis in critical care and cases of death (28-day, 75 day). By covering patients with different stages of sepsis, we can conclude that vitamins are not beneficial for different sepsis conditions.

Clinical indicators often indicate a decrease in vitamin C concentration in patients with sepsis [31]. But in our study, we discovered that the serum vitamin C level is not affected by sepsis exposure. We first extracted SNPs that were strongly associated with five different levels of sepsis of varying severity. Then, MR analysis was used to investigate whether sepsis is related to plasma vitamin C levels. Our result may not conflict with past studies. The reduction of ascorbic acid in sepsis patients may be caused by pleiotropic effects in biologic pathways associate with sepsis [32] and there was no direct link between sepsis and plasma vitamin C levels. For instance, vitamin C can scavenge reactive oxygen species (ROS) [33], septic shock patients have significantly depleted vitamin C levels likely due to increased metabolism resulting from the enhanced inflammatory response observed in septic shock. [31]. For vitamin D, clinical research results indicate that lower levels of vitamin D are associated with a higher likelihood of infection and greater disiease severty [34, 35]. Serum vitamin D deficiency may facilitate infections from Staphylococcus aureus and Clostridium difficile, leading to prolonged hospital stays and increased associated costs [36]. Vitamin D levels may be correlated with lower systemic inflammatory cytokines [26]. Some clinical study results suggest a correlation between vitamin D levels and APACHE II and SOFA scores. Vitamin D has pleiotropic regulatory effects, influencing immune function, endothelial cells, mucous membranes, as well as the metabolism of blood glucose and calcium. Vitamin D deficiency results in immune and metabolic dysfunction, contributing to adverse outcomes [37]. However, our study results show that the severity of sepsis is not causally related to vitamin D levels, and there is no genetic association for the susceptibility to sepsis based on vitamin D levels. We believe that, similar to vitamin C, there is no direct causal relationship between vitamin D levels and sepsis severity, this may also explain the current contradictions in clinical research findings.

Vitamin A, K and B vitamins tend to have no direct association with sepsis. Vitamin A is an antioxidant with anti-infection and anti-inflammatory effects and is associated with many inflammatory markers [38]. Vitamin A deficiency in sepsis patients may be triggered by increased metabolic demands and an increase in vitamin A and retinol-binding protein (RBP) excretion in the urine during severe infection [39]. A randomized controlled trial indicated that for septic adult patients, ICU days, ventilator hours, pressor agent usage, or mortality rate in 28 days were not significantly reduced by vitamin A treatment [40]. It is consistent with our study that shows vitamin A has no benefit in curing sepsis. For B vitamins, deficiencies in vitamin B12 and folic acid will adversely affect homocysteine metabolism and lead to hyperhomocysteinemia, which can worsen sepsis. When patients took standardized enteral nutrition and adequate B vitamins, a difference still existed between survivors and nonsurvivors regarding homocysteine concentration [41]. This suggests that vitamin B12 and folic acid may not be associated with hyperhomocysteinemia and worsening sepsis during this process. Another study also found no difference in vitamin B12 and folate concentrations between sepsis patients and healthy controls [42]. This is consistent with our finding that those two vitamins are not related with sepsis. Vitamin B6 is constantly being pointed out to have antioxidant and anti-inflammatory effects [43] in protecting peripheral organs. Vitamin B6 has been suggested to reduce oxidative stress in sepsis [44]. Nevertheless, our study points out that vitamin B6 has no therapeutic value for sepsis, and there is no valid correlation between the two. We did not find any randomized controlled trials directly investigating whether vitamin K can treat sepsis, and only a few literatures have reported the possible correlation between the two. Sepsis is often accompanied by coagulopathy [45]. Proteins Gas6 and protein S, which are vitamin K dependent, are known to activate TAM receptors, which have anti-inflammatory and coagulation properties [46]. However, these potential associations do not reveal whether vitamin K has a therapeutic effect on sepsis, and our study does not suggest a strong association.

We further investigated the role of vitamins in the current clinical practices and guidelines for sepsis. Despite the Surviving Sepsis Campaign (SSC) guidelines weakly recommending against intravenous vitamin C for adult patients with sepsis or septic shock (low-quality evidence) [47], we acknowledge the widespread interest in the use of vitamins in sepsis management. Observational studies suggest that early intravenous administration of vitamin C, when combined with corticosteroids and thiamine, is effective in preventing the gradual deterioration of organ function, including acute kidney injury, and reducing mortality in patients with severe sepsis and septic shock. While as emphasized by subsequent randomized controlled trials (RCTs) and meta-analyses [8, 48], our study results did not directly support a correlation between various vitamins and the risk or severity of sepsis. However, it does not negate the role of vitamins in sepsis, prompting us to pay attention to the indirect pathophysiological changes caused by vitamin levels. Future research needs to comprehensively consider the role of vitamins in sepsis. This should be accomplished through additional large-scale, RCTs to rigorously validate their effects, rather than being incorporated into clinical practice based solely on biological plausibility, low-quality studies, and presumed safety [49].

Our research has some advantages. Using mendelian randomization, we investigated the role of various vitamins in sepsis from a genetic standpoint for the first time. We simultaneously selected five kinds of sepsis of different severity from UK Biobank for research, which made our results more robust. The people studied were all European, reducing the bias caused by demographic stratification. Our results also do not contradict MR’s hypotheses. Some limitations exist in our study. MR studies have inherent limitations, including weak instrumental variables pleiotropy, low statistical power, canalization, population stratification [50]. Firstly, weak instrumental variables could introduce uncertainty in causal inferences, challenging the reliability of the study conclusions. Pleiotropy might introduce unknown confounding factors, making the results more complex and challenging to interpret. Low statistical power could result in the study’s inability to detect true effects, affecting the accuracy of the conclusions. Additionally, canalization and population stratification could introduce biases in the results, and it’s essential to carefully consider the impact of these factors when interpreting the study conclusions. We have tried to address these limitations in our study. Folate, vitamin A, B6, K, have only a few instrumental variables, although no associated bias was detected. Though sepsis of different severity was selected for our study, sepsis is a disease with remarkably heterogeneous condition [51]. Therefore, the selected patients with sepsis still need to further refined according to different Status of illness. Because GWAS data are only available from European populations, our results may not be applicable to all populations. Some isoforms of vitamins were not involved and need to be considered in subsequent studies.

## Conclusions

In conclusion, from the perspective of genetic association, our MR study did not find that vitamins (vitamin B6, B12, A, D, C, K and folate) could reduce the risk of sepsis. Sepsis also had no effect on plasma vitamin C levels in the body. More relevant randomized controlled trials need to be conducted to confirm the results.

### Electronic supplementary material

Below is the link to the electronic supplementary material.

Supplementary Material 1

## Data Availability

The UK Biobank data are accessible under application at https://www.ukbiobank.ac.uk/ (accessed date: 12 January 2022), and other all data described in our study are provided within this article.
