# Supplementary material for: Causal effects of genetically vitamins and sepsis risk: a two-sample Mendelian randomization study
Source: BMC Infect Dis. 2023 Nov 7;23:766. doi: 10.1186/s12879-023-08778-9 (PMC10629037; doi:10.1186/s12879-023-08778-9)
Supplement: Supplementary file 1 — Supplementary Material 1 [file 12879_2023_8778_MOESM1_ESM.docx]

**Supplementary information**

**Table S1.** Characteristics of vitamins-associated instrumental variants

| Exposure | SNP | Chr | Ea | Oa | EAF | Beta | SE | *P*-value | F |
| --- | --- | --- | --- | --- | --- | --- | --- | --- | --- |
| Vitamin B6 | rs4654748 | 1 | T | C | 0.5 | 1.45 | 0.28 | 8.30E-18 | 26.82 |
| Folate | rs652197 | 11 | C | T | 0.18 | 0.069 | 0.011 | 1.40E-12 | 39.35 |
| Folate | rs1801133 | 1 | G | A | 0.67 | 0.096 | 0.008 | 9.50E-53 | 144.00 |
| Vitamin B12 | rs2336573 | 19 | T | C | 0.03 | 0.32 | 0.007 | 8.40E-59 | 2089.80 |
| Vitamin B12 | rs1131603 | 22 | C | T | 0.06 | 0.19 | 0.017 | 4.90E-49 | 124.91 |
| Vitamin B12 | rs3742801 | 14 | T | C | 0.29 | 0.045 | 0.009 | 1.70E-13 | 25.00 |
| Vitamin B12 | rs2270655 | 4 | G | C | 0.94 | 0.066 | 0.018 | 2.20E-13 | 13.44 |
| Vitamin B12 | rs12272669 | 11 | A | G | 0.01 | 0.51 | 0.007 | 3.00E-09 | 5308.16 |
| Vitamin B12 | rs34324219 | 11 | C | A | 0.88 | 0.21 | 0.007 | 1.10E-111 | 900.00 |
| Vitamin B12 | rs34528912 | 11 | T | C | 0.04 | 0.17 | 0.021 | 2.10E-15 | 65.53 |
| Vitamin B12 | rs117456053 | 11 | G | A | 0.98 | 0.16 | 0.026 | 1.90E-09 | 37.87 |
| Vitamin B12 | rs602662 | 19 | A | G | 0.6 | 0.16 | 0.007 | 2.40E-139 | 522.45 |
| Vitamin B12 | rs1801222 | 10 | G | A | 0.59 | 0.11 | 0.007 | 3.30E-75 | 246.94 |
| Vitamin B12 | rs56077122 | 10 | A | C | 0.34 | 0.087 | 0.009 | 4.80E-21 | 93.44 |
| Vitamin B12 | rs41281112 | 13 | C | T | 0.95 | 0.17 | 0.02 | 8.90E-35 | 72.25 |
| Vitamin B12 | rs1141321 | 6 | C | T | 0.63 | 0.061 | 0.007 | 3.60E-26 | 75.94 |
| Vitamin A | rs10882272 | 10 | C | T | 0.378 | -0.087 | 0.01488 | 7.00E-15 | 34.18 |
| Vitamin A | rs1667255 | 18 | C | A | 0.369 | 0.09 | 0.01534 | 6.00E-14 | 34.42 |
| Vitamin C | rs6693447 | 1 | T | G | 0.551 | 0.039 | 0.006 | 6.25E-10 | 42.25 |
| Vitamin C | rs13028225 | 2 | T | C | 0.857 | 0.102 | 0.009 | 2.38E-30 | 128.44 |
| Vitamin C | rs33972313 | 5 | C | T | 0.968 | 0.36 | 0.018 | 4.61E-90 | 400.00 |
| Vitamin C | rs10051765 | 5 | C | T | 0.342 | 0.039 | 0.007 | 3.64E-09 | 31.04 |
| Vitamin C | rs7740812 | 6 | G | A | 0.594 | 0.038 | 0.006 | 1.88E-09 | 40.11 |
| Vitamin C | rs174547 | 11 | C | T | 0.328 | 0.036 | 0.007 | 3.84E-08 | 26.45 |
| Vitamin C | rs117885456 | 12 | A | G | 0.087 | 0.078 | 0.012 | 1.70E-11 | 42.25 |
| Vitamin C | rs2559850 | 12 | A | G | 0.598 | 0.058 | 0.006 | 6.30E-20 | 93.44 |
| Vitamin C | rs10136000 | 14 | A | G | 0.283 | 0.04 | 0.007 | 1.33E-08 | 32.65 |
| Vitamin C | rs9895661 | 17 | T | C | 0.817 | 0.063 | 0.008 | 1.05E-14 | 62.02 |
| Vitamin D | rs2282679 | 4 | T | G | 0.74 | 0.087 | 0.002964 | 2.40E-189 | 861.41 |
| Vitamin D | rs10741657 | 11 | A | G | 0.41 | 0.034 | 0.002687 | 1.10E-36 | 160.06 |
| Vitamin D | rs4944062 | 11 | T | G | 0.7 | 0.035 | 0.002921 | 4.30E-33 | 143.62 |
| Vitamin D | rs10745742 | 12 | T | C | 0.39 | 0.016 | 0.002747 | 5.70E-09 | 33.93 |
| Vitamin D | rs17216707 | 20 | T | C | 0.79 | 0.028 | 0.00351 | 1.50E-15 | 63.63 |
| Vitamin K | rs4645543 | 8 | T | C | 0.04 | -0.42 | 0.08 | 2.00E-27 | 27.56 |
| Vitamin K | rs2192574 | 2 | C | T | 0.11 | 0.28 | 0.06 | 1.82E-26 | 21.78 |

Abbreviations: SNP, single nucleotide polymorphism; Chr, chromosome; Ea, effect allele; Oa, non-effect allele; EAF, Effect allele frequency; SE, standard error of Beta; F, F statistics (F = beta^2^/se^2^).

**Table S2.** Characteristics of sepsis-associated instrumental variants

| Exposure | SNP | Chr | Ea | Oa | EAF | Beta | SE | *P*-value | F |
| --- | --- | --- | --- | --- | --- | --- | --- | --- | --- |
| Sepsis | rs11068069 | 12 | T | C | 0.1764 | 0.0830 | 0.0179 | 3.60E-06 | 21.47 |
| Sepsis | rs11980516 | 7 | T | G | 0.8241 | 0.0885 | 0.0181 | 9.50E-07 | 24.03 |
| Sepsis | rs12544445 | 8 | T | C | 0.1827 | -0.0855 | 0.0183 | 2.95E-06 | 21.85 |
| Sepsis | rs139409755 | 16 | T | C | 0.0409 | -0.1667 | 0.0347 | 1.55E-06 | 23.09 |
| Sepsis | rs147734876 | 5 | T | G | 0.0108 | -0.3154 | 0.0678 | 3.30E-06 | 21.63 |
| Sepsis | rs147793338 | 6 | A | G | 0.0302 | 0.1931 | 0.0422 | 4.80E-06 | 20.91 |
| Sepsis | rs150753765 | 3 | G | A | 0.0157 | -0.2721 | 0.0558 | 1.09E-06 | 23.76 |
| Sepsis | rs3851566 | 10 | G | A | 0.4439 | 0.0629 | 0.0137 | 4.63E-06 | 20.98 |
| Sepsis | rs4841254 | 8 | A | G | 0.6534 | -0.0684 | 0.0144 | 1.85E-06 | 22.74 |
| Sepsis | rs80054869 | 4 | C | T | 0.0121 | -0.3003 | 0.0635 | 2.27E-06 | 22.35 |
| SepsisCC | rs114583226 | 2 | A | C | 0.0426 | 0.5303 | 0.0982 | 6.73E-08 | 29.14 |
| SepsisCC | rs12644781 | 4 | T | C | 0.5588 | 0.2001 | 0.0395 | 4.02E-07 | 25.68 |
| SepsisCC | rs149106381 | 1 | A | G | 0.0158 | -0.7437 | 0.1608 | 3.72E-06 | 21.40 |
| SepsisCC | rs8137707 | 22 | T | C | 0.1280 | 0.2888 | 0.0589 | 9.22E-07 | 24.09 |
| SepsisU75 | rs10500376 | 16 | A | G | 0.0931 | -0.1084 | 0.0230 | 2.41E-06 | 22.24 |
| SepsisU75 | rs11240118 | 1 | A | G | 0.7139 | 0.0679 | 0.0148 | 4.69E-06 | 20.96 |
| SepsisU75 | rs114920411 | 6 | A | G | 0.0396 | -0.1686 | 0.0346 | 1.08E-06 | 23.78 |
| SepsisU75 | rs4713940 | 6 | A | G | 0.7941 | -0.0779 | 0.0165 | 2.54E-06 | 22.14 |
| SepsisU75 | rs76605533 | 9 | T | C | 0.0118 | 0.2872 | 0.0618 | 3.35E-06 | 21.60 |
| Sepsis28 | rs114724320 | 8 | T | C | 0.0253 | -0.4918 | 0.1069 | 4.24E-06 | 21.15 |
| Sepsis28 | rs145413915 | 3 | T | C | 0.0172 | 0.6646 | 0.1407 | 2.33E-06 | 22.30 |
| Sepsis28 | rs148818459 | 17 | T | C | 0.0104 | -0.7920 | 0.1676 | 2.29E-06 | 22.34 |
| Sepsis28 | rs17090050 | 18 | A | G | 0.2561 | -0.1748 | 0.0382 | 4.85E-06 | 20.89 |
| Sepsis28 | rs177999 | 18 | T | C | 0.1783 | 0.2015 | 0.0439 | 4.39E-06 | 21.09 |
| Sepsis28 | rs190351470 | 11 | T | C | 0.0201 | -0.5589 | 0.1205 | 3.49E-06 | 21.52 |
| Sepsis28 | rs34209645 | 2 | A | G | 0.4303 | 0.1590 | 0.0338 | 2.53E-06 | 22.14 |
| Sepsis28 | rs470236 | 11 | G | A | 0.8074 | 0.1975 | 0.0422 | 2.93E-06 | 21.86 |
| Sepsis28 | rs4787745 | 16 | A | G | 0.9224 | 0.2898 | 0.0625 | 3.49E-06 | 21.53 |
| Sepsis28 | rs62340396 | 4 | T | G | 0.0292 | -0.4626 | 0.0997 | 3.53E-06 | 21.51 |
| Sepsis28 | rs7155416 | 14 | T | G | 0.1125 | 0.2432 | 0.0530 | 4.38E-06 | 21.09 |
| Sepsis28 | rs77613868 | 3 | T | C | 0.0139 | -0.6736 | 0.1438 | 2.79E-06 | 21.95 |
| Sepsis28 | rs824453 | 2 | C | T | 0.1491 | 0.2231 | 0.0468 | 1.85E-06 | 22.74 |
| Sepsis28 | rs9955473 | 18 | C | T | 0.3147 | -0.1690 | 0.0362 | 3.02E-06 | 21.81 |
| Sepsis28CC | rs112291026 | 7 | G | A | 0.0334 | -1.0273 | 0.2220 | 3.72E-06 | 21.40 |
| Sepsis28CC | rs117022102 | 8 | C | T | 0.0196 | 1.3993 | 0.2862 | 1.01E-06 | 23.90 |
| Sepsis28CC | rs145913929 | 2 | C | T | 0.0267 | 1.2972 | 0.2525 | 2.80E-07 | 26.39 |
| Sepsis28CC | rs6684036 | 1 | T | G | 0.1936 | -0.4978 | 0.1002 | 6.77E-07 | 24.68 |
| Sepsis28CC | rs73790123 | 5 | G | T | 0.0377 | 0.9605 | 0.2080 | 3.89E-06 | 21.32 |
| Sepsis28CC | rs78078730 | 16 | T | C | 0.0276 | -1.1507 | 0.2425 | 2.08E-06 | 22.52 |
| Sepsis28CC | rs80135360 | 5 | A | G | 0.0187 | 1.4145 | 0.2873 | 8.49E-07 | 24.24 |

Abbreviations: SNP, single nucleotide polymorphism; Chr, chromosome; Ea, effect allele; Oa, non-effect allele; EAF, Effect allele frequency; SE, standard error of Beta; F, F statistics (F = beta2/se2); SepsisCC, Sepsis (critical care); SepsisU75, Sepsis (under 75); Sepsis28, Sepsis (28 day death); Sepsis28CC, Sepsis (28 day death in critical care).

**Table S3.** SNPs associated with potential confounders

| Trait | SNP | Confounder | *P*-value |
| --- | --- | --- | --- |
| Sepsis | rs139409755 | Mean corpuscular hemoglobin | 3.27E-06 |
| Sepsis | rs4841254 | Body mass index | 4.92E-09 |
| SepsisU75 | rs114920411 | Rheumatoid arthritis | 3.70E-30 |
| SepsisU75 | rs4713940 | Height | 2.44E-23 |
| Sepsis28CC | rs6684036 | Phlebitis and thrombophlebitis | 8.84E-07 |
| Sepsis28CC | rs73790123 | Cause of death: cerebrum, except lobes and ventricles | 1.26E-07 |

Abbreviations: SNP, single nucleotide polymorphism; SepsisU75, Sepsis (under 75); Sepsis28CC, Sepsis (28 day death in critical care).

**Table S 4.**Result of causal effects of vitamins on sepsis outcomes

| Exposure | Outcome | Method | *P*-value | OR | 95%CI | Q(Q_pval) | *P*-value  (pleiotropy) |
| --- | --- | --- | --- | --- | --- | --- | --- |
| folate | Sepsis | IVW-FE | 0.258 | 1.164 | 0.895,1.514 | 0.001(0.971) | NA |
|  | SepsisCC | IVW-FE | 0.093 | 1.907 | 0.898,4.050 | 1.796(0.180) | NA |
|  | SepsisU75 | IVW-FE | 0.474 | 1.099 | 0.849,1.421 | 0.047(0.829) | NA |
|  | Sepsis28 | IVW-FE | 0.248 | 1.459 | 0.769,2.771 | 0.018(0.892) | NA |
|  | Sepsis28CC | IVW-FE | 0.102 | 3.526 | 0.778,15.979 | 0.004(0.950) | NA |
| vitamin B6 | Sepsis | Wald ratio | 0.164 | 0.987 | 0.969,1.005 | NA | NA |
|  | SepsisCC | Wald ratio | 0.874 | 0.996 | 0.944,1.050 | NA | NA |
|  | SepsisU75 | Wald ratio | 0.100 | 0.985 | 0.967,1.003 | NA | NA |
|  | Sepsis28 | Wald ratio | 0.435 | 0.982 | 0.939,1.027 | NA | NA |
|  | Sepsis28CC | Wald ratio | 0.369 | 0.953 | 0.857,1.059 | NA | NA |
| vitamin B12 | Sepsis | IVW-FE | 0.454 | 0.975 | 0.914,1.041 | 13.547(0.331) | 0.939 |
|  | SepsisCC | IVW-FE | 0.927 | 0.991 | 0.822,1.195 | 9.353(0.673) | 0.385 |
|  | SepsisU75 | IVW-FE | 0.462 | 0.976 | 0.916,1.041 | 12.446(0.411) | 0.960 |
|  | Sepsis28 | IVW-FE | 0.770 | 1.024 | 0.873,1.201 | 11.090(0.521) | 0.804 |
|  | Sepsis28CC | IVW-FE | 0.571 | 0.897 | 0.616,1.306 | 7.770(0.803) | 0.477 |
| vitamin A | Sepsis | IVW-FE | 0.953 | 0.993 | 0.797,1.238 | 0.635(0.426) | NA |
|  | SepsisCC | IVW-FE | 0.793 | 0.919 | 0.489,1.727 | 0.0001(0.992) | NA |
|  | SepsisU75 | IVW-FE | 0.366 | 0.905 | 0.730,1.123 | 0.568(0.451) | NA |
|  | Sepsis28 | IVW-FE | 0.244 | 1.375 | 0.804,2.352 | 0.495(0.482) | NA |
|  | Sepsis28CC | IVW-FE | 0.982 | 1.015 | 0.287,3.587 | 0.010(0.922) | NA |
| vitamin D | Sepsis | IVW-RE | 0.558 | 0.861 | 0.522,1.420 | 12.379(0.015) | 0.965 |
|  | SepsisCC | IVW-FE | 0.613 | 0.810 | 0.359,1.831 | 4.313(0.365) | 0.856 |
|  | SepsisU75 | IVW-RE | 0.527 | 0.864 | 0.550,1.358 | 10.573(0.032) | 0.663 |
|  | Sepsis28 | IVW-FE | 0.772 | 0.903 | 0.451,1.806 | 2.212(0.697) | 0.771 |
|  | Sepsis28CC | IVW-FE | 0.077 | 0.230 | 0.045,1.176 | 5.826(0.213) | 0.330 |
| vitamin K | Sepsis | IVW-FE | 0.380 | 0.955 | 0.860,1.059 | 1.891(0.169) | NA |
|  | SepsisCC | IVW-FE | 0.289 | 0.851 | 0.631,1.147 | 0.003(0.957) | NA |
|  | SepsisU75 | IVW-FE | 0.933 | 1.004 | 0.907,1.112 | 0.597(0.440) | NA |
|  | Sepsis28 | IVW-FE | 0.063 | 0.786 | 0.610,1.013 | 2.267(0.132) | NA |
|  | Sepsis28CC | IVW-FE | 0.053 | 0.554 | 0.305,1.007 | 0.930(0.335) | NA |
| vitamin C | Sepsis | IVW-FE | 0.505 | 1.049 | 0.911,1.208 | 9.408(0.400) | 0.113 |
|  | SepsisCC | IVW-FE | 0.604 | 1.113 | 0.743,1.667 | 14.404(0.109) | 0.442 |
|  | SepsisU75 | IVW-FE | 0.848 | 0.987 | 0.859,1.133 | 6.081(0.732) | 0.674 |
|  | Sepsis28 | IVW-RE | 0.625 | 0.889 | 0.553,1.427 | 17.186(0.046) | 0.969 |
|  | Sepsis28CC | IVW-FE | 0.856 | 0.927 | 0.409,2.101 | 10.823(0.288) | 0.828 |

Abbreviations: IVW-RE, inverse-variance weighted multiplicative random effects; IVW-FE, inverse-variance weighted multiplicative fixed-effects; OR, odds ratio; 95%CI, 95% confidence interval of odds ratio; SepsisCC, Sepsis (critical care); SepsisU75, Sepsis (under 75); Sepsis28, Sepsis (28 day death); Sepsis28CC, Sepsis (28 day death in critical care); Q(Q_pval), Cochran’s Q statistic (P-value of Cochran’s Q statistic) of heterogeneity test; P-value(pleiotropy), P-value of pleiotropy test

**Table S 5.** Result of causal effects of sepsis on plasma vitamin C concentration outcomes

| Outcome | Exposure | Method | *P*-value | OR | 95%CI | Q(Q_pval) | *P*-value  (pleiotropy) |
| --- | --- | --- | --- | --- | --- | --- | --- |
| plasma vitamin C concentration | Sepsis | IVW-FE | 0.612 | 1.018 | 0.949,1.092 | 3.689(0.815) | 0.772 |
|  | SepsisCC | IVW-FE | 0.415 | 1.013 | 0.981,1.047 | 1.982(0.576) | 0.328 |
|  | SepsisU75 | IVW-FE | 0.317 | 1.062 | 0.944,1.196 | 3.103(0.212) | 0.925 |
|  | Sepsis28 | IVW-FE | 0.166 | 1.015 | 0.994,1.037 | 13.860(0.384) | 0.053 |
|  | Sepsis28CC | IVW-FE | 0.945 | 0.999 | 0.985,1.015 | 0.583(0.965) | 0.591 |

Abbreviations: IVW-RE, inverse-variance weighted multiplicative random effects; IVW-FE, inverse-variance weighted multiplicative fixed-effects; OR, odds ratio; 95%CI, 95% confidence interval of odds ratio; SepsisCC, Sepsis (critical care); SepsisU75, Sepsis (under 75); Sepsis28, Sepsis (28 day death); Sepsis28CC, Sepsis (28 day death in critical care); Q(Q_pval), Cochran’s Q statistic (P-value of Cochran’s Q statistic) of heterogeneity test; P-value(pleiotropy), P-value of pleiotropy test
